# Effect of post-space irrigation with acid solutions on bond strength and dentin penetrability using a self-adhesive cementation system

**DOI:** 10.4317/jced.58029

**Published:** 2021-06-01

**Authors:** Aryvelto-Miranda Silva, Cristiane-de Melo Alencar, Fernanda-Ferreira-de Albuquerque Jassé, Victor-Feliz Pedrinha, Joissi-Ferrari Zaniboni, Andréa-Abi-Rached Dantas, Edson-Alves de Campos, Milton-Carlos Kuga

**Affiliations:** 1 Department of Restorative Dentistry, Araraquara School of Dentistry, São Paulo State University (UNESP), Araraquara, SP, Brazil; 2 School of Dentistry, Federal University of Para (UFPA), Belém, PA, Brazil; 3 Department of Restorative Dentistry, Bauru School of Dentistry, University of Sao Paulo (USP), Bauru, SP, Brazil

## Abstract

**Background:**

The aim of this study was to evaluate the effects of surface treatments with 1% peracetic acid (PA), solution containing 17% EDTA (SmearClear, Kerr Endodontics), solution containing a combination of 17% EDTA with 2% chlorhexidine (QMix, Dentsply Sirona) on the post-space root dentin compared to 2.5% sodium hypochlorite (NaOCl) on bond strength and resin tags length in dentin.

**Material and Methods:**

Forty human-canine roots were endodontically treated and the post space was prepared. The specimens were randomised into four groups (n = 10): control – irrigation with 2.5% NaOCl solution, PA – irrigation with 1% PA, SmearClear – irrigation with SmearClear solution, and QMix – irrigation with QMix solution. The fibre posts were cemented using a self-adhesive resin system (Relyx U200, 3M ESPE). After six months, the specimens were cross-sectioned and subjected to push-out and confocal laser microscopy tests. One-way ANOVA and Tukey’s tests were used to analyse the data (α= 0.05).

**Results:**

PA and QMix presented the highest bond strength values compared to the other groups (*p*< 0.05). There was no significant difference between the resin tags length in dentin by the groups (*p* = 0.75).

**Conclusions:**

Irrigation of the post space with 1% PA and QMix showed a positive clinical impact on the adhesion between the fiber post and root dentin. However, these materials had no influence on resin tags length in dentin by self-adhesive resin cement.

** Key words:**Fiber post, adhesive cementation, self-adhesive resin cement, root dentin, irrigating solutions.

## Introduction

Endodontically treated teeth are characterised by loss of coronal structure and decreased mechanical strength of the intraoral chewing forces (1). In a recent meta-analysis, fibre posts were associated with higher overall survival rates than metal posts when used to restore endodontically treated teeth with extensive coronal destruction (2). Several characteristics favour the clinical choice of fibre posts; they include the more conservative removal of dentin, the aesthetic advantages, and greater treatment agility since their cementation does not require a laboratory step (3,4).

Self-adhesive resin cement, such as RelyX U200 (3M ESPE, Saint Paul, MN, USA), was introduced to reduce the sensitivity of the conventional three-step adhesive technique and prevent errors arising from the clinical protocol. Conventional and self-adhesive resin types of cement have similar effectiveness (5). However, self-etching resin cement requires careful irrigation of the post space with solutions that do not interfere with the bond strength and increase its penetrability in the dentinal tubules. Residues can impair adhesion to root dentin. Thus, satisfactory adhesion of the posts cementation is dependent on the safe removal of the smear layer (6).

Chelating solutions are an effective approach in removing debris and the smear layer (7). QMix (Dentsply Sirona Endodontics, Tulsa, OK, USA) is a commercial product indicated for irrigation and intra-canal cleaning; it is designed to remove the inorganic smear layer and disinfect the root canal system. The chemical composition of QMix combines the chelating properties of ethylenediaminetetraacetic acid (EDTA) and the substantive and antimicrobial properties of chlorhexidine gluconate (8). In addition, it is composed of a mixture of an antimicrobial agent from bisbiguanide, saline, and a surfactant (9). It was previously demonstrated that QMix, as a cleaning solution, showed less decalcification and erosion than 17% EDTA (10). However, further studies should be carried out to ascertain this.

SmearClear (Kerr Endodontics, Orange, CA, USA) is a commercial product which was introduced to aid the removal of the smear layer. It is a 17% EDTA solution combined with cationic (cetrimide) and anionic surfactants to improve its antibacterial effectiveness and clinical performance (7). However, evidence on its positive impact on dentin adhesion is controversial (7,11).

Peracetic acid (PA) is a promising alternative to obtain the disinfection of the root canal system and dissolve the smear layer safely (12). It demonstrates sporicidal, bactericidal, virucidal, and fungicidal activities at low concentrations. It breaks down into safe by-products and oxygen, and it reduces smear layer formation as effectively as EDTA (13). A recent study showed that 1% PA alone provided a reduction in microhardness and roughness of the root canal dentin similar to what was observed for EDTA and sodium hypochlorite (NaOCl). However, 1% PA caused less dentin erosion than EDTA and sodium hypochlorite (12). Studies on the use of 1% PA as a single endodontic irrigating solution in the preparation of the post space are limited. Furthermore, various endodontic irrigating solutions should be thoroughly compared to provide consistent evidence.

Therefore, the objective of this ex vivo study was to evaluate the effects of surface treatments with 1% peracetic acid (PA), solution containing 17% EDTA (SmearClear, Kerr Endodontics), solution containing a combination of 17% EDTA with 2% chlorhexidine (QMix, Dentsply Sirona) on the post-space root dentin compared to 2.5% sodium hypochlorite (NaOCl) on bond strength and resin tags length in dentin by self-adhesive cementation system. The null hypotheses of this study were as follows: (a) H01 – endodontic irrigation solutions used for post-space preparation do not affect the bond strength between root dentin and fibre posts cemented with self-adhesive resin, and (b) H02 – endodontic irrigation solutions used in post-space preparation have no influence on resin tags length in dentin by self-adhesive cementation system.

## Material and Methods

-Sample Preparation

This study followed the ethics recommendations for human research and was approved by the Local Research Ethics Committee. After adequate education on the risks, methods, and objectives of this study, all research participants signed the Free and Informed Consent Form in accordance with the Declaration of Helsinki. Forty canines with similar root anatomy and extracted for periodontal reasons were selected. Periapical radiographs were acquired in the mesiodistal and buccolingual directions to select teeth with single canals. All teeth were evaluated with the aid of a stereomicroscope (SMX800, Nikon Co., NY, USA) under 20× magnification. Those which presented immature apexes, root caries, root fractures, cracks, lacerations, sharp curvatures, canal calcifications, or endodontic treatment were excluded. The specimens were stored in a 0.1% thymol solution at 4 °C until use. After being rinsed with distilled water, teeth were cross sectioned using a diamond disk under water cooling (Isomet 2000; Buehler Ltd., Lake Buff, IL, USA). All roots were standardised to a length of 17 mm.

A single surgeon prepared the instrumentation of the root canals. Canal patency was performed with a 10 size K-file (Dentsply Maillefer, Ballaigues, Jura-Nord Vaudois, Switzerland) and Path-File instruments 1, 2, and 3 (Dentsply Maillefer, Ballaigues, Jura-Nord Vaudois, Switzerland). The working length (WL) of the canals was standardised to 16 mm. The root canals were prepared with the aid of the Universal ProTaper System (Dentsply Maillefer, Ballaigues, Jura-Nord Vaudois, Switzerland). Initially, S1, SX, and S2 were used for the cervical and middle thirds of the roots prepare. Subsequently, S1, S2, F1, F2, F3, F4, and F5 files were consecutively used in the WL. Finally, the canals were irrigated with 5 mL of 2.5% NaOCl (Asfer, São Caetano do Sul, SP, Brazil). The foraminal openings were sealed with composite resin to prevent the extrusion of NaOCl during the irrigation procedures. After canal mechanic preparation, the irrigation was performed with 5 mL of 17% EDTA (Biodinâmica, Ibiporã, PR, Brazil) for 3 minutes. In sequence, the specimens were rinsed with 5 mL of 2.5% NaOCl and completed with 5 mL of saline solution using Navitip needles (Ultradent Products Inc., South Jordan, UT) taken up to 3 mm short of the WL.

All the root canals were dried with F5 paper points (Dentsply Maillefer, Ballaigues, Jura-Nord Vaudois, Switzerland) and filled using the single cone technique (F5 gutta-percha point, Dentsply Maillefer) and an epoxy resin-based sealer (AH Plus; Dentsply De Trey, Konstanz, Baden-Württemberg, Germany). The endodontic sealer was mixed following the manufacturer’s instructions and placed at the WL using a 400-rpm Lentulo spiral (Dentsply Maillefer, Ballaigues, Jura-Nord Vaudois, Switzerland) for 5 seconds. The remainder gutta-percha in the coronal portion was removed using a flame-heated plugger and the access cavity was sealed (Coltosol, Coltene, Rio de Janeiro, RJ, Brazil). All roots were stored in artificial saliva (Faculdade de Ciências Farmacêuticas-UNESP, Araraquara, SP, Brazil) at 37oC for 7 days. Afterward, they were placed in plastic matrices as described by a previous study (14).

The partial remotion of the root canal filling was performed after 24h. For this, was used the Largo 1 to 4 drill sequence (Dentsply Maillefer, Ballaigues, Jura-Nord Vaudois, Switzerland), associated with saline solution irrigation, at a length of 11 mm. Post-space preparation was performed using the White Post #2 burr (White Post DC, FGM, Joinville, SC, Brazil) at 11 mm. Periapical radiographs were performed to certify the root filling remotion.

-Treatment with irrigation solutions

Samples were randomised into 4 groups (n = 10) based on the irrigation solution used during post-space preparation: 2.5% NaOCl (control group), 1% PA (Peresal, Profilática, Araucária, PR, Brazil), SmearClear (Kerr, Orange, CA, USA), and QMix (Dentsply Tulsa Dental, Tulsa, OK, USA). The post space was irrigated and aspired with 5 mL of the tested solution for 1 minute, and the solution remained inside the root canal for 3 minutes in Group 2.5% NaOCl and Group 1% PA, 1 minute in Group SmearClear, and 2 minutes in Group QMix, following the manufacturer’s recommendations. Subsequently, the post spaces were rinsed with 5 mL of saline solution and dried with 0.48-mm-diameter capillary tips (Capillary Tips; Ultradent, South Jordan, UT, USA) and F5 paper points.

Fibre posts #2 (White Post DC, FGM, Joinville, SC, Brazil) were cleaned with 70% ethyl alcohol, coated with silane (Prosil, FGM, Joinville, SC, Brasil), and put out to dry for 1 minute to allow solvent evaporation.

-Fibre post cementation

To provide fluorescence in the confocal laser microscopy assessment, the Rhodamine B dye (Sigma-Aldrich, St. Louis, MO, USA), in the proportion of 0.1% (m/m), was incorporated in the self-adhesive resin cement (RelyX U200, 3M ESPE, St. Paul, USA). The manufacturer’s recommendations were followed for handling resin cement, which was inserted into the post-space with the aid of Automix tips (3M ESPE, St. Paul, USA). The post was positioned in the post space by manual pressure. The excess resinous cement was removed with a micro brush and cured for 40 s using an LED light-curing unit (LED Bluephase, Ivoclar Vivadent, Schan, AL, Liechtenstein) with an intensity of 1200 mW/cm2. Light curing was performed so that the light guide tip of the light-curing unit remained perpendicular to the post. In sequence, specimens were stored in distilled water at 37oC for 7 days.

-Bond strength and failure mode evaluation 

After the storage period, three slices (thickness 2.0mm ±0.1mm) were obtained from each root. For this, a diamond disk under water cooling in a cutting machine (Isomet, Buehler Ltd, Lake Bluff, IL, USA) was used. Cervical, middle, and apical slices were obtained at 1, 5, and 8 mm, respectively, from the cervical root surface. Irregularities generated during the sectioning procedures were removed with 1.200 water-wet sandpapers (3M ESPE, Saint Paul, MN, USA) in a polishing machine (Buehler, Lake Bluff, Illinois, USA).

The push-out test was conducted in a universal testing machine (EMIC, São José dos Pinhais, PR, Brazil) at a crosshead speed of 0.5 mm/min. The slices were inserted into a metal device with a central hole (Ø = 3 mm) larger than the diameter of the canal. The coronal face of the specimen was placed face down and a metallic cylinder (Ø extremity = 0.8 mm) advanced a load onto the pole from the apical to the coronal direction. The bond strength values (σ) were obtained with the following formula - MPa: σ = F/A, where F and A represents the load for breaking the sample (n) and the bonded area (mm2), respectively. The bonded interface area was determined applying the following formula: A = πg (R1 + R2); π = 3.14, g = slant height, R1 = smaller base radius, and R2 = larger base radius. The slant height was determined with the following formula was used: g2 = [h2 + (R2 -R1)2], where h = section height. R1 and R2 were deduced from the internal diameters of the minor and major bases, respectively. The measurement of the diameters and section heights were performed with a digital caliper (Starrett 727, Starrett, Itu, SP, Brazil).

Dentin slices were analysed under a stereomicroscope (Zeiss Stemi SV6) at 20× magnification to categorise the failure modes, following the classification: Type 1 – adhesive failure at the fibre post/cement interface; Type 2 – cohesive failure inside the cement; Type 3 – adhesive failure at the dentin/cement interface; and Type 4 – mixed failure occurs in combination (15). In addition, failure mode was assessed using a confocal laser microscope (LEXT OLS4100; Olympus, Shiniuki-ku, Tokyo, JP) to illustrate each pattern.

-Resin tags length in dentin

After the push-out test, intratubular cement penetration was examined using an Olympus FluoView Confocal Laser 1000 microscope (Olympus Corporation, Tokyo, Japan). Cervical, middle, and apical samples were assessed. The rhodamine B is absorbed and emitted in wavelengths 540 nm and 494 nm, respectively. For dentin samples analysis, a 10× oil lens at 100× magnification was used. The image depth was 70 µm and the dimension was 800 × 800 pixels. Adobe Photoshop software (Adobe Systems, San Jose, CA, USA) was used for image analysis as previously described (16). Initially, the overall area of the image was obtained. Next, the lasso tool was used to outline and measure the canal area. By subtracting these values, the dentine area was obtained. Thus, the sealer-impregnated dentin area was outlined with the same tool, and the percentage of sealer penetration was calculated.

-Statistical analysis 

SPSS statistical software was used for analysis (SPSS 12.0; SPSS Inc., Chicago, IL, USA). The normality of the data was verified using the Shapiro Wilk test, which confirmed normal distribution of the data; W = 0.842 and W = 0.832 for bond strength and intratubular cement penetration, respectively. Thus, these outcomes were analysed using one-way ANOVA and Tukey’s tests (α = 0.05).

## Results

One-way ANOVA indicated significant effects of the irrigant solutions in bond strength for all post-space thirds (*p*<0.05). In the analysis with all thirds grouped, 1% PA and QMix groups had the highest mean bond strength values (*p*<0.05). In the cervical and middle thirds, 1% PA and QMix promoted higher bond strength values than 2.5% NaOCl and SmearClear (*p* < 0.05). In the apical third of the post space, QMix presented superiority regarding 1% PA (*p* < 0.05), groups with the highest bond strength values. The lowest bond strength was found in Group 2.5% NaOCl (*p* < 0.05). The bond strength means values with standard deviations for all groups in each post-space third are presented in Table 1.

**Table 1:**
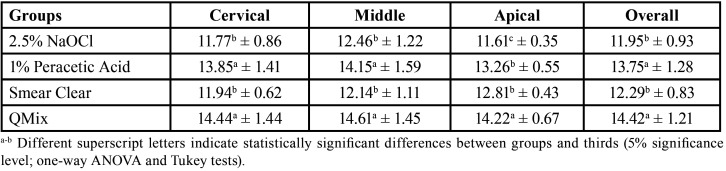
Mean and standard deviation of the bond strength values (MPa) for all groups in each post-space third.

**Figure 1:**
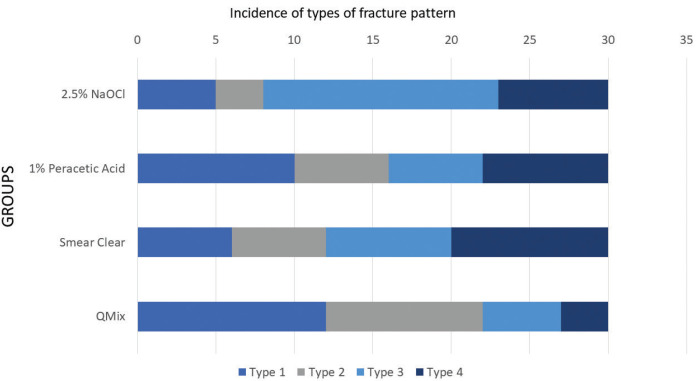
Incidence of the type of fracture pattern in each group: Type 1 – adhesive failure at the fibre post/cement interface; Type 2 – cohesive failure inside the cement; Type 3 – adhesive failure at the dentin/cement interface; and Type 4 – mixed failure occurs in combination.

**Figure 2:**
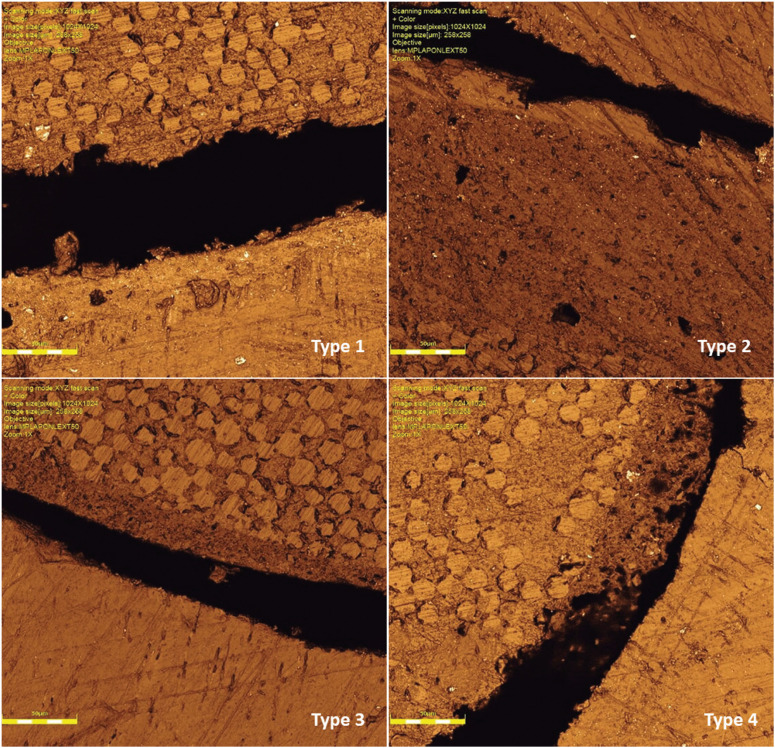
Ilustration of the fracture pattern by means of laser confocal microscopy: Type 1 – adhesive failure at the fibre post/cement interface; Type 2 – cohesive failure inside the cement; Type 3 – adhesive failure at the dentin/cement interface; and Type 4 – mixed failure occurs in combination.

Adhesive failures in the cement interface with dentin (N=34) and post (N=33) were the most observed, followed by mixed failures (N = 28) and cohesive failures of the cement (N = 25). Also, numerous dentin-cement interface failures occurred in 2.5% NaOCl group (Fig. 1). Figure 2 illustrates each failure pattern.

**Figure 3:**
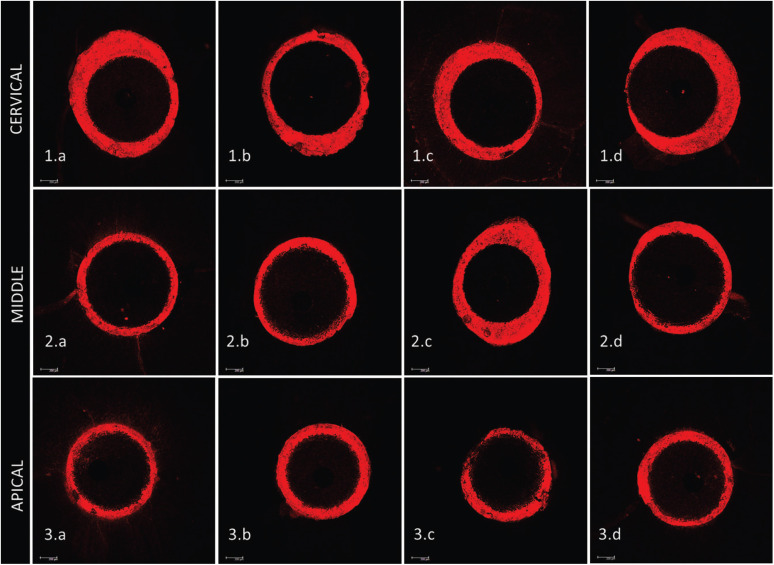
Photomicrographs of the cervical, middle and apical thirds based on surface treatment: 1. Cervical third: (1.a) - 2.5% NaOCl; (1.b) - 1% Peracetic acid; (1.c) - 17% EDTA; (1.d) - QMix solution; 2. Middle third: (2.a) 2.5% NaOCl; (2.b) 1% Peracetic acid; (2.c) - 17% EDTA; (2.d) - QMix solution; 3. Apical third: (3.a) 2.5% NaOCl; (3.b) 1% peracetic acid; (3.c) - 17% EDTA; (3.d) - QMix solution. Scale: 200µm.

After the assessment of intratubular cement penetration, one-way ANOVA revealed no differences between the groups, irrespective of the post-space third evaluated (*p* = 0.85) (Table 2, Fig. 3).

**Table 2:**
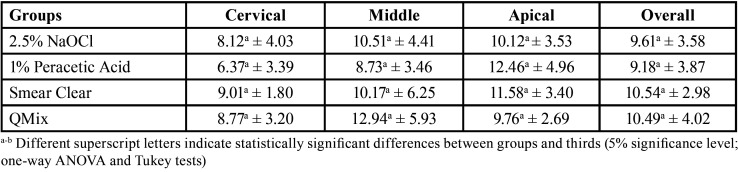
Mean and standard deviation (%) of intratubular resin cement (U200) penetration after final irrigation of post space.

## Discussion

Considering the need for further studies related to this topic, the present study investigated the effect of different intracanal irrigants on the bond strength between root dentin and fibre posts cemented with self-adhesive resin cement and the intratubular cement penetration after post-space irrigation with different solutions. Given the results, the first null hypothesis was rejected. The irrigant used for post-space irrigation influenced the bond strength between the root dentin and fibre posts cemented with self-adhesive resin cement. On the other hand, the second null hypothesis was confirmed. The irrigant used for post-space irrigation did not affect intratubular cement penetration.

The RelyX U200 manufacturer recommends that NaOCl be the solution of choice for irrigating the post-space preparation before the fiber post cementation; however, the criteria used to choose this irrigant to remain unclear. Two recent studies have shown that post-space irrigation with 2.5% NaOCl did not facilitate the best bond strength when compared to other alternative treatments (6,17). However, Barreto *et al*. (7) found higher bond strength values when saline and 2.5% NaOCl were used, and they recommended these solutions for post-space cleaning before post cementation when using self-adhesive cement. It must be highlighted that in the cited study, the authors performed passive ultrasonic irrigation (PUI). In the present study, PUI was not performed. Furthermore, the irrigation solutions were used according to the manufacturer’s recommendations, and thus, the actual effect of the solutions on root dentin was assessed.

The literature reported that the effect of NaOCl on organic dentin compounds may promote collagen degradation and negatively affect the resistance of the fibre posts to root dentin (18). In addition, Zhang *et al*. stated that when dentin was exposed to high concentrations of NaOCl for long periods, its bonding and flexural strength were impaired (19). Therefore, the duration of dentin exposure to NaOCl and the concentration of NaOCl were negatively correlated with adhesive strength (15,19). In the present study, an intermediate concentration of NaOCl (2.5%) was used and the exposure duration was 3 minutes; this duration was enough to promote post-space cleaning and collagen degradation since the values of bond strength were low in all evaluated post-space thirds.

The QMix solution and PA showed the highest bond strength in the cervical and middle thirds of the post space; in the apical third, only QMix showed the best adhesive bonding results. This may be attributed to the chemical composition of QMix and its mechanism of action and effect on dentin. QMix contains EDTA, chlorhexidine, and a detergent in its formulation; this guarantees its low surface tension and increases its wettability. It also facilitates its flow in the root canal in contact with the smear layer and the underlying dentin (20); this activity is probably due to the mixture of chlorhexidine and the detergent (21), resulting in the highest bond strength.

In the literature, there are reports of studies which aimed to evaluate the effect of active irrigation using ultrasound and neodymium: yttrium-aluminum-garnet (Nd: YAG) laser to remove the smear layer and debris after post-space preparation (7,22). Some studies have shown better cleaning of the root canal walls when ultrasonic and Nd: YAG activation was performed, mainly in association with chelating solutions (23,24). On the other hand, some authors did not observe an improvement in the removal of the smear layer and debris when active irrigation was performed (22,25). This study evaluated only the chemical effect promoted by the chelating solutions, and it did not include another variable such as PUI or Nd: YAG laser.

Among the various descaling agents available, PA may be the best alternative to other chelating solutions. PA contains and releases acetic acid, which is a weak chelating agent capable of reducing the smear layer with the same effectiveness as EDTA, for example (12,13). To the authors’ best knowledge, only one study is available in the literature on the use of PA before post-cementation and its impact on the bond strength fibre posts cemented with self-adhesive cement (6). However, 1% PA had greater cytotoxic potential than 2.5% NaOCl, which is a disadvantage of this solution (26). Considering this, further investigations and comparisons between different solutions are encouraged to increase the evidence on this issue. A previous study showed that SmearClear does not facilitate better smear layer removal than EDTA alone (27), which is consistent with the findings of this study since SmearClear and 2.5% NaOCl presented statistically similar bond strength values. The duration of contact between the solutions and dentin recommended by the manufacturer was maintained to prevent erosion on the dentin surface.

Failure patterns were variable in all the groups. An interesting finding was that Group NaOCl had adhesive failures at the cement-dentin interface in 50% of the specimens. This may indicate an inadequate level of chemical bonding between the self-adhesive cement and root dentin caused by the degradation of collagen promoted by NaOCl (18). It may be argued that Group NaOCl should have undergone final irrigation with 17% EDTA. However, this protocol aimed to create a negative control with a remaining smear layer, since some studies have reported that EDTA dissolves the sparse “ghost mineral layer” in collagen and exposes and irreversibly damages the underlying dentin when it is used as a solvent for inorganic matter after preparation of the root canal with NaOCl (28). Therefore, we can hypothesise that an irrigation protocol which alternates between NaOCl and EDTA causes more deleterious effects on dentinal tissues than unique solution protocols with disinfection and chelating activities, which could jeopardise our results. This is the reason for choosing the 2.5% NaOCl solution.

Kuga *et al*. (29) observed no differences in the associations between NaOCl and acid solutions over the depth of penetration into the root dentin. Confocal laser scanning microscopy (CLSM) is often used in endodontics to determine the degree of adaptation and dentin wall and dentinal tubule penetrations of the root canal filling (30). According to the CLSM images of the present study, no correlation was observed between cement penetration and bond strength, even in the 2.5% NaOCl group, which can be related to the duration of exposure and the renovation-dependent character of this solution, as mentioned above. However, EDTA and NaOCl when associated with ultrasound activation can be promising solutions for cleaning root canals before cementing a fibre post using self-adhesive resin cement (7). Additional studies are necessary to fully understand the relationship between the depth of the penetration of the self-adhesive resin cement and bond strength. In addition, further *in vivo* studies are required to assess the long-term effect of different post-space treatments on the bond strength between adhesive materials and root dentin.

## Conclusions

Considering the limitations of this *ex vivo* study, the PA at 1% and QMix facilitated the highest bond strength, while 2.5% NaOCl facilitated the lowest. The failure patterns varied, with high adhesive failures at the dentin-cement interface for NaOCl and SmearClear root surface treatment. The dentinal tubule penetration of the cement was not affected by the solution used to rinse the post space.
